# Wild Gazelles of the Southern Levant: Genetic Profiling Defines New Conservation Priorities

**DOI:** 10.1371/journal.pone.0116401

**Published:** 2015-03-11

**Authors:** Lia Hadas, Dalia Hermon, Amizor Boldo, Gal Arieli, Ron Gafny, Roni King, Gila Kahila Bar-Gal

**Affiliations:** 1 The Koret School of Veterinary Medicine, The Robert H. Smith Faculty of Agriculture, Food and Environment, The Hebrew University of Jerusalem, Rehovot, Israel; 2 DNA and Forensic Biology Laboratory, Division of Identification and Forensic Science, Israel Police, National Headquarters, Jerusalem, Israel; 3 Supervision and Enforcement Division, Israel Nature and Parks Authority, Jerusalem, Israel; 4 Science and Conservation Division, Israel Nature and Parks Authority, Jerusalem, Israel; University of Illinois at Urbana-Champaign, UNITED STATES

## Abstract

The mountain gazelle (*Gazella gazelle*), Dorcas gazelle (*Gazella Dorcas*) and acacia gazelle (*Gazella arabica acacia*) were historically abundant in the southern Levant, and more specifically in Israel. Anthropogenic and natural changes have caused a rapid decline in gazelle populations, raising concerns about their conservation status and future survival. The genetic profile of 111 wild gazelles from Israel was determined based on three regions of mitochondrial DNA (control region, *Cytochrome b* and 12S ribosomal RNA) and nine nuclear microsatellite markers. Genetic analysis of the mountain gazelle population, the largest known population of this rare species, revealed adequate diversity levels and gene flow between subpopulations. Nevertheless, ongoing habitat degradation and other human effects, such as poaching, suggest the need for drastic measures to prevent species extinction. Dorcas gazelles in Israel displayed inbreeding within subpopulations while still maintaining considerable genetic diversity overall. This stable population, represented by a distinctive genetic profile, is fragmented and isolated from its relatives in neighboring localities. Based on the genetic profile of a newly sampled subpopulation in Israel, we provide an alternative hypothesis for the historic dispersal of Dorcas gazelle, from the Southern Levant to northern Africa. The small acacia gazelle population was closest to gazelles from the Farasan Islands of Saudi Arabia, based on mitochondrial markers. The two populations did not share haplotypes, suggesting that these two populations may be the last remnant wild gazelles of this species worldwide. Only a dozen acacia gazelles survive in Israel, and urgent steps are needed to ensure the survival of this genetically distinctive lineage. The genetic assessments of our study recognize new conservation priorities for each gazelle species in the Southern Levant.

## Introduction

**Fig 1:**
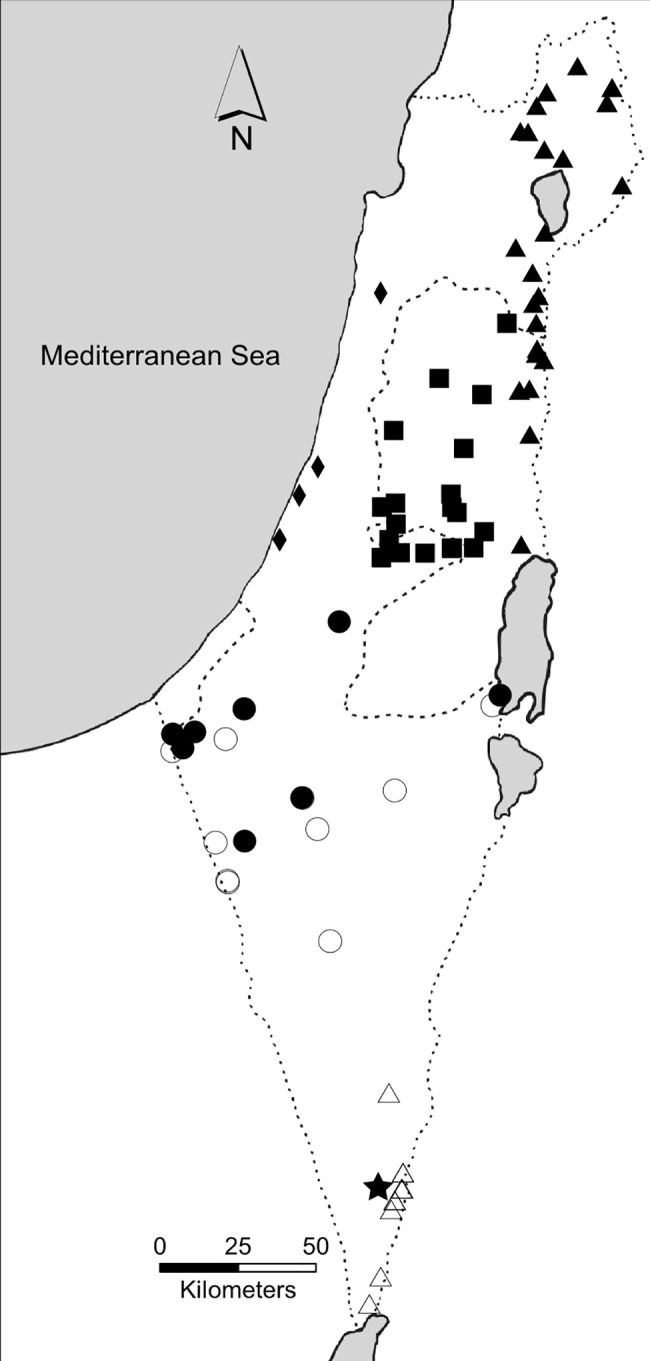
Sampling sites of gazelles across the Southern Levant. The division by species and geographic regions is indicated by the different icons. Shaded icons represent mountain gazelles: triangles represent the Northern subpopulation, squares represent the Central subpopulation, diamonds represent the Coastal subpopulation and circles represent the Western Negev subpopulation. Dorcas gazelles are represented by unshaded icons: circles represent the Negev subpopulation and the triangles the Arava subpopulation. Acacia gazelles were sampled only from the Arava (shaded star).

The genus *Gazella* (Family: *Bovidae*, Subfamily: *Antilopinae*) is distributed widely across Africa, the Middle East and Asia [1]. Prior to the domestication of livestock, the most frequently hunted species in the southern Levant was the mountain gazelle (*Gazella gazella*) [2,3]. In the last century, hunting has remained a major threat to gazelle populations, together with recent climatic and anthropogenic changes (rapid growth of the human population, increased infrastructure, intensive agriculture). These have resulted in the fragmentation of gazelle populations throughout their ranges. In Israel three species of *Gazella* are extant: mountain gazelles, the most common gazelle in Israel, is found from the Golan Heights in the North throughout Central Israel, the Jordan Rift Valley and the Northern Negev (IUCN classification VU-vulnerable); Dorcas gazelles (*Gazella dorcas*) distributed from the Northern Negev southwards (IUCN classification VU); and the endemic acacia gazelles (presumed *Gazella arabica* subspecies) with a very limited home range in the Arava Valley, and with most confined to a nature reserve for protection (Fig. 1). Mountain gazelles have been reported as extinct in Syria, and possibly extinct in Lebanon and Jordan, making the population in Israel regionally unique [4]. The Dorcas gazelle has a wider distribution and is found mainly across North Africa. The acacia gazelles in the Arava Valley constitute the only known surviving population of this Arabian gazelle subspecies. Annual gazelle surveys in Israel conducted by the Israel Nature and Parks Authority (INPA) indicate a continuous trend of population declines among all species, especially mountain gazelle [5]. The main factors contributing to this alarming decline in Israel are illegal hunting, predation, road kills and habitat fragmentation [6]. As of 2013 INPA reported population sizes of approximately 3000 mountain gazelles, 800 Dorcas gazelles and 12 acacia gazelles. In 2012 50 acacia gazelles were counted but since then their number has declined to ∼12 due to severe habitat disturbance by floods and predation (personal communication, R. Talbi).

The decline in *Gazella* numbers is evident worldwide, and there is an urgent need for researchers to determine their conservation units and to describe phylogenetic relationships among species [1,7,8]. In 2010 Wronksi *et al*. [8] reported the existence of two reciprocally monophyletic lineages within *Gazella gazella* (a Northern clade represented by samples from the Golan Heights, and a Southern clade represented by gazelles from the Arabian Peninsula and the Arava valley) based on two mitochondrial markers: control region and *cytochrome b*. These results were further supported by Lerp et al. using bi-parentally inherited nuclear microsatellite markers, including also gazelles from Central Israel [7]. Both of these studies, as well as a recent genetic analysis of the *Gazella arabica* lectotype [9] and a morphological study [10], have divided *Gazella gazella* into two distinct genetic lineages: *Gazella gazella* (mountain gazelles—the northern clade, original type locality) and *Gazella arabica* (Arava valley and the Arabian Peninsula). Therefore the mountain gazelles found in Israel are recognized as a distinct species.

Extensive ecological and behavioral studies have been conducted on the gazelle populations of Israel [11,12,13,14,15,16,17]. However, no detailed research was ever undertaken to examine the genetic structure of these populations. The goal of this study was to assess the genetic structure and genetic diversity of the three gazelle species in Israel. This information will be vital for conservation management, in assessing extinction risks and identifying distinct management units [18,19,20].

## Materials and Methods

### Ethics Statement

No review from the ethics committee was required, as our research work did not involve any direct manipulation or disturbance of animals. Blood samples were collected by and under the permit of the Israel Nature and Parks Authority. Access to all sampling locations was not restricted. No specific permissions were required.

### Samples

Tissue and blood samples of 111 wild gazelles (68 mountain gazelles, 32 Dorcas gazelles and 11 acacia gazelles) were collected from different locations across the distribution of the species in Israel, from 2005 to 2012 (Fig. 1). Tissues were sampled from road kills and blood samples were collected from wild animals receiving veterinary treatment from INPA rangers and veterinarian (S1 Table), thus no animal was harmed for the purposes of this study.

### Sampling Locations

Each sample was assigned to a subpopulation based on its geographic origin (S1 Table, Fig. 1). Mountain gazelles were divided into four subpopulations: 1. Northern: Golan Heights, the eastern slopes of the Galilee and the Jordan Rift Valley down to the Dead Sea; 2. Central: the mountainous terrain of the West Bank, Jerusalem and the Judean foothills (Shfela); 3. Coastal: along the Mediterranean coastline; 4. Western Negev (WNegev): the southernmost subpopulation living around the 200 mm precipitation line (Fig. 1). Dorcas gazelles were divided to two subpopulations—the Negev subpopulation, sampled along the western parts of the Negev desert, and the Arava subpopulation in the southern part of the Jordan Rift Valley (Fig. 1). Acacia gazelles were sampled only in one location in the Arava. Since 2006 roughly all of the known acacia gazelles have resided in a designated protected area, the Hai-Bar Yotvata nature reserve, which they share with Dorcas gazelles (Fig. 1).

### DNA Extraction

DNA extraction from blood samples was carried out using the Chelex-100 (Bio-Rad) protocol [21] or the AccuPrep Genomic DNA extraction kit (BioNeer). DNA from tissue samples was extracted using the guanidinium thiocyanate method followed by a silica-based (GuSCN) purification method [22,23] or Chelex-100.

### DNA Amplification

#### Mitochondrial DNA

For each sample three mitochondrial regions were amplified: 12s rRNA (167bp) [24], control region (CR) (328bp) (F: 3′-GCCATAGCCCCACTATCAA CAC-5′, R: 3′-TTTTGACTTAAATGTGCTATGTACG-5′) and a region of the *Cytochrome B* gene together with tRNA-Thy and tRNA-Pro (CytB) (790bp) (F: 3′- ATGAGGACAAATATCAT TTTGAGG-5′, R: 3′-GTTTAAGTAGAATTTCAGCTTTGGGT-5′). The latter two primer sets were designed using the online primer design software Primer3Plus [25]. PCR amplifications were conducted using the high-fidelity AmpliTaq Gold DNA Polymerase (Applied Biosystems) to minimize polymerase errors. Reactions had a final volume of 25μl with 1.25 units *Taq*-Gold, 3.5mM MgCl_2_, 0.3mM dNTPs, 0.4μM of each primer and 1x buffer. Touchdown PCR cycling conditions were as follows: initial denaturation at 95°C for 10 min followed by a total of 35 cycles of 15 sec at 95°C, 30 sec annealing for 3 cycles each at 60°C, 58°C, 56°C, 54°C, 52°C and 50°C, and 17 cycles at 48°C, and elongation at 72°C for 45 sec; after the 35 cycles there was a final extension step of 10 min at 72°C.

PCR products were visually examined on a 1.5% agarose gel. PCR products of the expected size were purified using Exonuclease I—shrimp alkaline phosphatase (HDV Pharmacia) prior to Sanger sequencing. Sequencing reactions were performed on an ABI PRISM 3730xl DNA Analyzer at the Center for Genomic Technologies at the Hebrew University of Jerusalem. Each sample was Sanger sequenced for the sense and the anti-sense strands of each marker and a consensus sequence was determined. CR and Cytb sequences have been submitted to GenBank (accession numbers KM523344-KM523547), 12S sequences are available as supporting information (S1 File).

#### Nuclear DNA

Nuclear microsatellite markers were adapted from cattle, sheep and goats, 10 loci in total (S2 Table [26,27,28,29,30,31,32,33,34,35]) Each forward primer was fluorescently labeled and multiplexes were designed using Multiplex Manager 1.0 [36]. Reactions had a final volume of 15μl with 0.625 units *Taq*-Gold, 3mM MgCl_2_, 0.3mM dNTPs, 0.4μM each primer and 1x buffer. PCR cycling conditions were as follows: initial denaturation at 95°C for 10 min followed by a total of 35 cycles of 15 sec at 95°C, 30 sec annealing at the temperature specific for each multiplex (S2 Table), and elongation at 72°C for 45 sec, after 35 cycles there was a final extension step of 10 min at 72°C. The efficiency of the amplification was assessed on a 2% agarose gel and the labeled PCR products were detected on an ABI PRISM 3730xl DNA Analyzer at the Center for Genomic Technologies, The Hebrew University of Jerusalem, Israel. To ensure uniformity and lack of allele dropout, ten samples were genotyped more than once. Each microsatellite locus was validated by direct sequencing on an ABI PRISM 3730xl DNA Analyzer.

### Data Analyses

#### Sanger Sequence Alignments and Diversity Measures

Sequence chromatograms were visually inspected using Sequencher 5.0 software (Genecodes, USA) for ambiguities and errors. Final consensus sequences were aligned using Geneious Pro 5.6.6 (BioMatters, New Zealand). Published sequences of gazelles from localities outside of Israel were obtained from GenBank, including those published by [1,8,9,37]. To assess the level of population differentiation, nucleotide diversity (π) and haplotype diversity (Hd) were calculated for the CR and Cytb fragments using DNASP v5 [38]. AMOVA and F_st_ calculations were executed in Arlequin 3.5 [39]. Sequences of the 12S fragment were primarily used for validation of species identification (mainly of the road kills), as gazelles are known to harbor intraspecific morphological differences. Due to the low variation in the 12S fragment it was not used in population differentiation analyses.

#### Phylogenetic Reconstructions

A maximum likelihood reconstruction of the phylogeny of mountain gazelles, acacia gazelles and Dorcas gazelles from Israel was performed using MEGA 5.1 software [40] with 1000 bootstrap replicates, based on the concatenated sequence (three mitochondrial fragments). The optimal model for the dataset (General Time Reversible model, Gamma distributed with Invariant sites (GTR+G+I, 5 discrete gamma categories) was determined via Modeltest incorporated in the above software. To infer intraspecific phylogenies among the different mitochondrial haplotypes we applied the median-joining algorithm [41] on the CR and Cytb datasets using the software Network 4.6 (fluxus-engineering.com). As the biggest dataset was available for the CR fragment, we used BEAST v1.8.0 [42] for the Bayesian phylogenetic reconstruction of the CR data and for estimation of divergence time. Analysis employed the same substitution model (GTR+G+I), Yule tree model, uniform priors and an uncorrelated lognormal relaxed molecular clock model with *a priori* information on the mean substitution rate per year (0.15 per million years) [37]. The different species were defined as taxa groups and two tMRCAs were set as priors: mountain and Arabian gazelles [mean 1.295 [7]] and the entire dataset [mean 2.1 [1]]. The performance of the BEAST runs was tested using Tracer v1.5 [43]. Following convergence, the resulting trees were combined using TreeAnnotator (part of the BEAST v1.8.0 package), to compute a maximum clade probability tree with a burn-in of 10% and posterior probability limit of 0.5. The tree was viewed in FigTree v1.4 [44].

#### Population Structure

Genotypes were scored by eye with GeneMapper (Applied Biosystems). Genotyping data was screened with MICROCHECKER version 2.2.3 to detect null alleles and heterozygote deficiency. Arlequin 3.5 software was used to test for Hardy-Weinberg equilibrium (HWE) and linkage disequilibrium (LD) in the microsatellite data. Genetic diversity measures (number of alleles, effective number of alleles, observed (H_o_) and expected (H_e_) heterozygosity and fixation index (F)) and measures of genetic distance between populations (F_st_ and Dest) were calculated using GenAlEx 6.5b3 [45]. The allele frequency spectrum (AFS) was used to check for signs of long term demographic changes [46].

The nuclear genetic partitioning was examined using STRUCTURE 2.3.4 [47] by inferring the most probable number of genetic clusters. The number of clusters (K) was assumed between one and 20 (*i*.*e*., K = 1 to K = 20) and each run was repeated five times. We used a burn-in period of 10,000 followed by 2,000,000 Markov chain Monte Carlo (MCMC) steps for each run, and the admixture model with the option of correlated allele frequencies was chosen. The same analyses were conducted without a priori definition of populations and with sampling locations as prior information (LOCPRIOR option). The analyses were carried out for the complete dataset and also separately for the mountain gazelles and for the Dorcas gazelles, to detect more subtle genetic structures. To verify that the results were not affected by null alleles we also utilized the recessive alleles option. To test for hybrids between the different species we conducted an additional analysis with the no admixture model for the complete dataset. Structure Harvester [48] was used to implement the Evanno method [49] for the STRUCTURE analyses results, to infer the best supported K using the ∆K statistics. Grouping of the samples was also carried out by Population Assignment Test and Principal Coordinates Analysis (PCoA) using GenAlEx.

## Results

All 111 samples were successfully Sanger sequenced for all three mitochondrial regions (total of 1286 base pairs for each sample). Quality control excluded 17 sequences from further analysis due to poor sequencing chromatogram quality. In addition, 99 nuclear microsatellite profiles were determined (59 mountain gazelles, 29 Dorcas gazelles and 11 acacia gazelles). Out of the 10 microsatellite loci, one locus (HAUT27) exhibited inconsistent amplifications and thus was removed from the analyses. For each species MICROCHECKER detected no signs of allele dropout but identified heterozygote deficiency suggesting that null alleles may be present at several loci which may lower the power of several of the analyses. Significant deviations from HWE (α = 0.05) were detected in each species, however, the results of both MICROCHECKER and HWE were not consistent across subpopulations for any loci, therefore all loci were included in the final analyses. Significant LD was found only for one combination of loci out of 36 combinations in Dorcas gazelles and acacia gazelle populations after Bonferonni correction.

### Genetic Relationships among Species

#### Mitochondrial Markers

We created three mitochondrial sequence alignments to be used for analyses: alignment I: a concatenated 1286 bp mtDNA fragment (12S, CR, Cytb), a total of 94 sequences from Israel obtained in this study (54 mountain gazelles, 29 Dorcas gazelles and 11 acacia gazelles); alignment II: 607 bp of the Cytb gene region consisting of 158 mtDNA sequences (105 this study and 53 published [1,37]); alignment III: 200 bp of CR utilizing 224 mtDNA sequences (99 this study and 125 published [8,9,37]). The latter two alignments represent samples from Israel and other localities in the Near East and North Africa. Three specimens initially (morphologically) identified as acacia gazelles were genetically identified as Dorcas gazelles using the 12S gene fragment. One sample, Gd13, was initially identified as a Dorcas gazelle, however our analyses (mtDNA and microsatellites) revealed it had Dorcas gazelle nuclear DNA (microsatellites) and acacia gazelle mtDNA (all three gene fragments). This sample was included in the phylogenetic analyses but excluded from the genetic diversity analyses.

**Fig 2:**
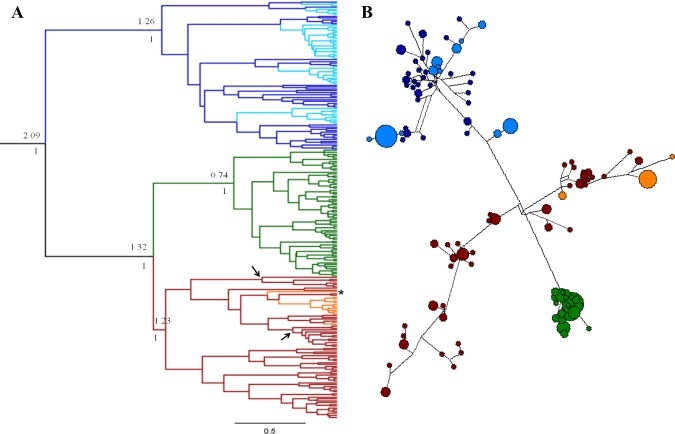
Genetic relationships among gazelles from the Southern Levant and other localities. 2a. Bayesian phylogenetic reconstruction. The phylogenetic analysis is based on sequences obtained from 230 individuals, 200bp of the control region, representing populations from the Southern Levant (this study, [8,37]) and from neighboring localities [8,37]. Numbers above the nodes represent node age in MYA while numbers below the nodes represent the posterior probability. Mountain gazelles are represented by the green color; Dorcas gazelles from Israel are represented by the light blue color; Dorcas gazelles from northern Africa are represented by the darker blue color; acacia gazelles are represented by the orange color and Arabian gazelles are represented by the red color. Gd13 is marked with an asterisk. Clades of the Arabian gazelles from the Farasan Islands are marked with an arrow at the node. 2b. Median-joining haplotype network. Size of the circle is proportional to the frequency of the haplotype. Color legend is the same as for the phylogenetic tree.

A phylogenetic analysis of all samples from our study (alignment I) exhibited strong support (P>0.95) for separation into three lineages, with a closer relationship between the mountain gazelles and acacia gazelles (Fig. S1 in S2 File). All three lineages were significantly differentiated from each other based on the F_st_ values ranging from 0.75 to 0.78 (Table 1). The differentiation of Israeli gazelle populations into three lineages was further supported by the Bayesian phylogenetic reconstruction and the median haplotype network analyses representing the gazelle populations from Israel together with gazelles from other localities (Fig. 2). The results of the Bayesian phylogeny and the CR haplotype network identified four main clusters: mountain gazelles, Dorcas gazelles and two Arabian gazelle clusters (Fig. 2). Mountain gazelles from Israel clustered tightly and separately from the other samples, reflecting their uniqueness and a low genetic diversity (Fig. 2). Dorcas gazelles from Israel clustered closely with the African Dorcas gazelles, as expected, however they did not share any haplotypes (Fig. 2). Arabian gazelles fell into two clades: one clade included all of the Arabian gazelles from the Farasan Islands (located off the western shore of Saudi Arabia) clustering with eight captive and three wild Arabian gazelles, as well as all acacia gazelles from Israel. No haplotypes were shared between acacia gazelles and Arabian gazelles (Fig. 2). The second clade comprised of captive gazelles from various locations on the Arabian Peninsula and two specimens from the wild, including one from the Sinai desert and one from Oman [8,37] (Fig. 2).

**Table 1:** Pairwise differences among populations of *Gazella* in Israel.

	**Mountain gazelles**	**Dorcas gazelles**	**Acacia gazelles**
**Mountain gazelles**	-	0.776	0.78
**Dorcas gazelles**	**0.155** [0.340]	-	0.752
**Acacia gazelles**	**0.268** [0.537]	**0.293** [0.412]	-

Microsatellites F_st_ (bold) and Dest (in brackets) are given below the diagonal, mitochondrial F_st_ are given above the diagonal. All values are significant at α = 0.05

Analysis of the CR between the acacia gazelles from Israel and Arabian gazelles from the Farasan Islands showed high F_st_ values (0.37). Similar levels of differentiation were found between the Farasan Islands Arabian gazelles and Arabian gazelles from the mainland of the Arabian Peninsula (0.39). Comparison of the acacia gazelles from Israel to mainland Arabian gazelles indicates significant genetic differentiation (Cytb F_st_ = 0.49; CR F_st_ = 0.51) indicating that they form distinct populations and potentially could be classified as different species.

#### Nuclear Markers

All microsatellite loci were polymorphic in both Dorcas and mountain gazelles with a mean of eight alleles per locus (Table 2). Given the small population size, the same microsatellite markers exhibited low polymorphism among acacia gazelles (less than three alleles per locus) with one locus (TGLA122) found to be monomorphic.

**Table 2:** Estimates of genetic diversity for the species of *Gazella* in Israel and their subpopulations. Based on a 1286bp mitochondrial fragment and nine microsatellite loci.

**Species**	**mtDNA**				**Microsatellites**					
	**N**	**H**	**π**	**Hd**	**N**	**Na**	**Np**	**Ho**	**He**	**F**
**Dorcas gazelle** ^a^	**28**	**10**	**0.0159**	**0.802**	**28**	**7.778**	**3.556**	**0.502**	**0.676**	**0.244**
Arava	14	6	0.0119	0.604	16.556	6.444	1.333	0.484	0.654	0.254
Negev	11	7	0.0170	0.909	9.444	5.333	0.667	0.548	0.654	0.119
**Mountain gazelle**	**54**	**21**	**0.0047**	**0.937**	**55.556**	**8.778**	**4.444**	**0.543**	**0.616**	**0.087**
Northern	22	16	0.0055	0.974	25.778	7.333	1.667	0.580	0.597	0.006
Central	21	13	0.0055	0.952	18.111	6.000	0.333	0.538	0.614	0.088
Coast	4	3	0.0009	0.833	3.556	2.889	0.111	0.435	0.459	0.075
Western Negev	7	6	0.0039	0.952	8.111	4.333	0.444	0.500	0.544	0.030
**Acacia gazelle** ^a^	**11**	**2**	**0.0051**	**0.327**	**10.444**	**2.556**	**0.333**	**0.382**	**0.353**	**−0.031**

N number of samples; H number of haplotypes; π nucleotide diversity; Hd haplotype diversity; Na number of different alleles; Np number of alleles unique to a single population; Ho observed heterozygosity; He expected heterozygosity; F fixation index [(He—Ho) / He = 1—(Ho / He)].

^a^ Excluding Gd13.

**Fig 3:**
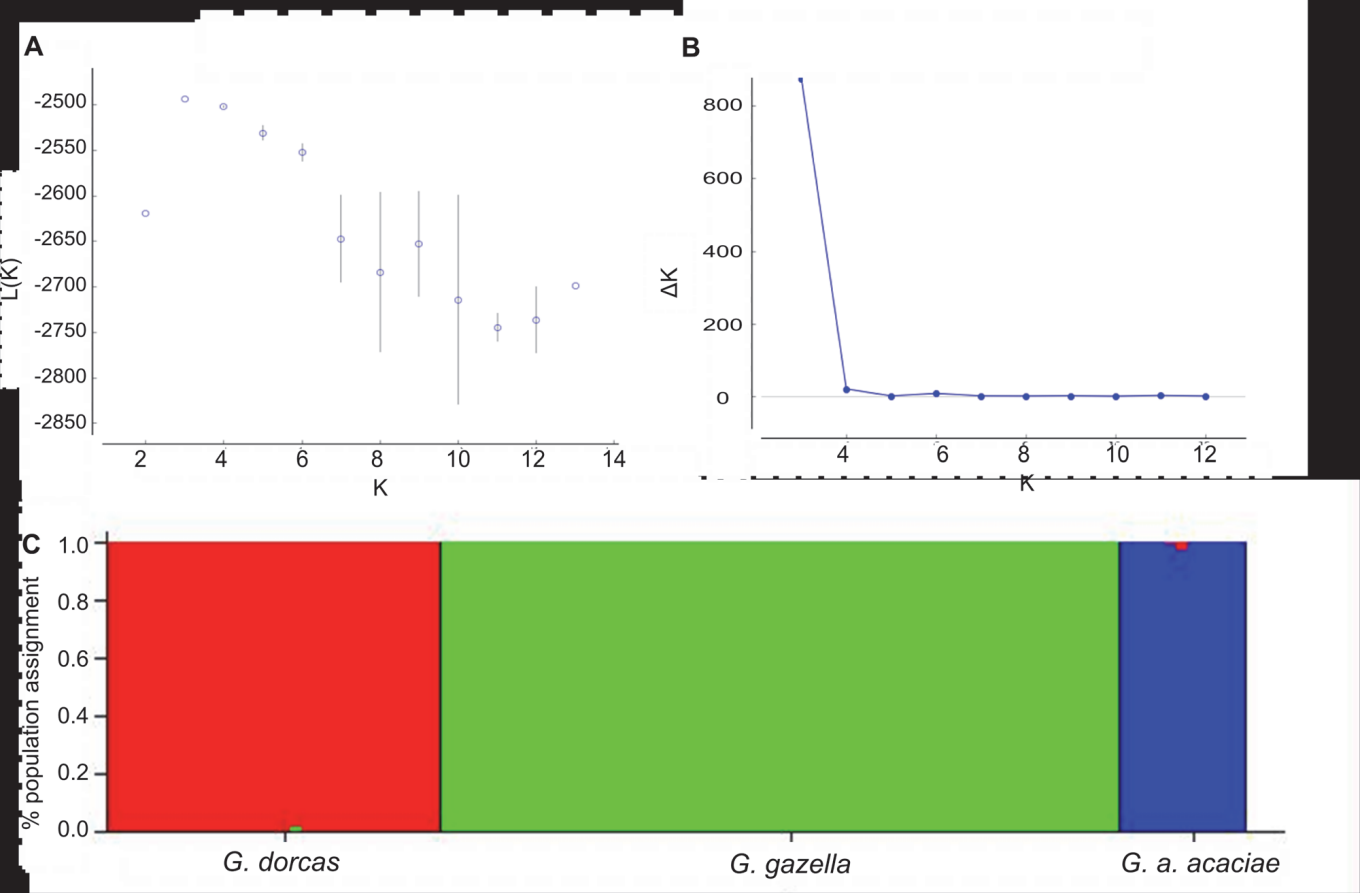
Bayesian clustering analyses for the three gazelle species in Israel. The most probable number of genetic clusters across three gazelle species in Israel using the STRUCTURE program with the recessive alleles option. (A) Mean L(K)±SD over 5 runs per K as a function of K. (B) ∆K (Evanno et al. 2005) as a function of K. (C) Population assignments to inferred genetic clusters at K = 3.

Nuclear genetic differentiation indices supported the separation of the samples into the same three lineages as shown by the mtDNA markers, F_st_ (0.15 to 0.3) and Dest (0.34 to 0.53) values were all high and significant (Table 1). The uppermost level of genetic structure of the entire dataset (99 samples from Israel genotyped by nine loci) was examined with STRUCTURE and divided the specimens into two clusters (K = 2) based on the standard admixture model and the Evanno method (∆K statistics): one cluster representing the Dorcas gazelles, and another representing the mountain and acacia gazelles. This grouping of mountain and acacia gazelles could reflect their relatedness as shown in the mtDNA phylogenetic analysis (Fig. 2 and Fig. S1 in S2 File). The second K level suggested 5 clusters: one cluster representing the acacia gazelles, two clusters representing mountain gazelles and two clusters of Dorcas gazelles (Fig. 3). The partition of the Dorcas and mountain gazelles into two clusters (each) was statistically significant using the genetic distance index (with Fst values greater than 0.07) however it did not correlate with optional variables such as geography. When analyzed with the LOCPRIOR option, recessive alleles option or no admixture model, the dataset was divided into three clusters, each species by itself (Figs. S4-S6 in S2 File). The same results were obtained with population assignment test and PCoA analysis (Fig. S7 in S2 File).

### Genetic Diversity within Species

#### Mountain gazelles

Mitochondrial diversity: All subpopulations of this species displayed low levels of nucleotide diversity, especially at the Cytb gene region, but relatively high haplotype diversity (Table 2). The lowest level of genetic diversity was found in the Coastal subpopulation, in which expected and observed heterozygosity (not affected by sample size) were also lowest (Table 2). Pairwise F_st_ between subpopulations reflected little or differentiation (Fst<0.04), with only one significant result supporting moderate differentiation (Fst = 0.10) between the WNegev subpopulation and the Northern subpopulation, the two geographically most distant subpopulations (S3 Table). Different geographic divisions of subpopulations were compared (Golan, Galilee, Jordanian rift Valley, Samarian, Judea, Coast and Western Negev—in different combinations), however each comparison gave the same result of little differentiation.

Phylogenetic trees based on the mtDNA alignment I (1286bp) placed the majority of mountain gazelle specimens into two supported clades that did not correspond to geographic origin (Fig. S1 in S2 File). Three individuals did not fall within these clades and were positioned as basal to the rest of the samples, each with unique haplotypes. One specimen originated from central Israel while the other two were from the Northern subpopulation.

Microsatellites diversity: The STRUCTURE analysis of the mountain gazelle microsatellite data identified three partitions (K = 3) using two different settings (∆k = 14.03, 8.5 for the standard and LOCPRIOR setting respectively) (Fig. S2A in S2 File). The geographic origin and the mtDNA haplotype of the samples did not correlate with the microsatellite clustering. F_st_ values also indicated mostly low or insignificant differentiation between the geographic subpopulations, with values lower than 0.07. Moderate differentiation was supported (significant) only between two neighboring subpopulations—the WNegev and Coastal (Fst = 0.10). The observed and expected heterozygosity values were similar in most subpopulations with fixation index values implying random mating among the subpopulations (F = 0.006–0.088) (Table 2). AFS revealed mutation drift equilibrium in the mountain gazelles. Overall, the various analyses using mitochondrial and nuclear markers indicate that the mountain gazelle comprise one population.

#### Dorcas gazelles

Based on mtDNA analyses two highly supported clades were identified among the Dorcas gazelle in Israel (Fig. S1 in S2 File). One clade was comprised of individuals from both the Arava and the Negev (Fig. S1 in S2 File). Haplotypes in this clade were also distinct from those of other Dorcas gazelles in the CR Network analysis (Fig. 2). The second clade was further divided into three clusters, with weak bootstrap support: one included only specimens from the Negev and the other two groups were predominantly specimens from the Arava (Fig. S1 in S2 File). A significant F_st_ value (0.133) was estimated between the two Dorcas geographic subpopulations (Negev, Arava) supporting a moderate differentiation.

**Fig 4:**
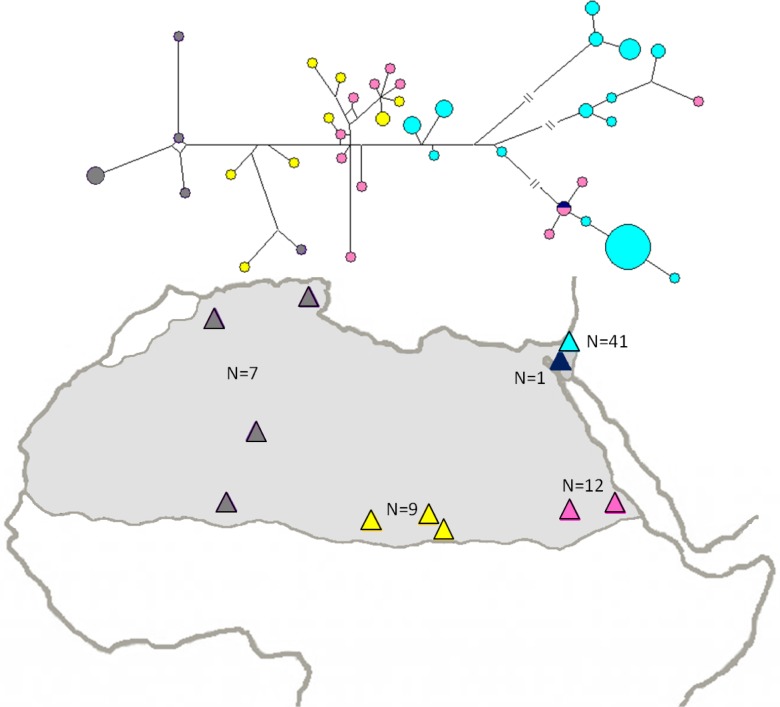
Median-joining haplotype network showing the relationships among Dorcas gazelle populations across the species range. Range of Dorcas gazelle distribution is shown on map in light grey, based on IUCN and published literature [37,60]. Triangles represent sampling locations, colors represent geographic grouping of samples (after [37]): Dark grey represents west, yellow represents south-central, pink represents south-east, dark blue represents Sinai and light blue represents Israel (our data). Haplotype network is based on concatenation of a 607bp fragment of CytB and a 200bp fragment of the CR, for a total of 70 individuals. Size of the circles is proportional to the frequency of the haplotype and the circle colors match geographic groupings.

High variation was found within the Dorcas gazelle population from Israel, as signified by the haplotype diversity (Hd) and nucleotide diversity (π) values (Table 2). For the Negev subpopulation genetic diversity was higher than in the Arava subpopulation (Table 2). Even when compared to other Dorcas populations [37,50], the population from Israel exhibited high diversity (Table 2). A haplotype network calculated based on the CytB and CR sequences illustrated a geographic separation of haplotypes (Fig. 4). Comparison of the Dorcas gazelle population from Israel to other geographic localities in northern Africa revealed that the closest relatives to Dorcas gazelles from Israel are individuals from Sudan (Fig. 2) with significant F_st_ estimate values (0.12 (CR) and 0.21 (Cytb)) (S4 Table).

Microsatellites diversity: Unlike the mtDNA markers, the geographic division of the two Dorcas gazelle subpopulations was less apparent in the microsatellite analyses, which revealed low differentiation between the Negev and the Arava subpopulations (Fst = 0.02). The observed heterozygosity (Ho) was lower than the expected heterozygosity (He) values by at least 0.1, which may indicate sex-biased dispersal or low gene flow between the subpopulations (Table 2). In addition, the fixation index values for the Negev (F = 0.119) and Arava (F = 0.254) subpopulations may suggest a higher possibility of inbreeding within the Arava (Table 2). The genetic structure (standard model k = 3; LOCPRIOR k = 6) found no clear clustering of individuals, suggesting that all individuals belong to one population (Fig. S3 in S2 File). As with the mountain gazelles, the AFS revealed mutation drift equilibrium within the Dorcas gazelles.

#### Acacia gazelles

Genetic diversity of the acacia gazelles was markedly lower than that of other Arabian gazelles. The low genetic diversity is characteristic of an enclosed and severely bottlenecked population such as this one, and was detected using both mitochondrial and nuclear DNA markers. Within the acacia gazelle clade, eight samples clustered tightly together and three samples diverged from this group, one of them the product of mitochondrial introgression (Gd13) (Fig. S1 in S2 File). A haplotype network of the CR showed the same clustering within the acacia gazelles (Fig. 2). Furthermore, comparison of the acacia gazelles to the subpopulation of Dorcas gazelles that shares the same geographic habitat (Arava valley) indicated a strong genetic differentiation with significant and high F_st_ values (mitochondrial Fst = 0.81, nuclear Fst = 0.3) (S3 Table). STRUCTURE (Fig. S6 in S2 File) and PCoA (Fig. S7 in S2 File) analyses identified two acacia gazelles as carrying alleles recognized as of Dorcas gazelle origin in two of the microsatellite markers (OarFCB48 and BM4505). The acacia sample size is relatively small and other markers did not harbor Dorcas gazelle associated alleles. In addition, the possibility of the natural occurrence of the Dorcas like alleles in markers OarFCB48 and BM4505 in the acacia gazelle population cannot be excluded. Therefore the two individuals were not identified as hybrids. A distorted allele distribution was shown for the acacia gazelle population in the AFS, once again reflecting the bottleneck in the history of the population.

## Discussion

Our study of wild gazelles from the Southern Levant (n = 111) revealed their uniqueness compared to other populations of the same species worldwide. In all three species, the populations in Israel harbored distinguishing haplotypes (Fig. 2), which were not shared by specimens from other localities, implying their genetic isolation and distinctiveness.

### Influences of Ancient Events on the Populations’ Genetic Structure

Environmental forces during the Pleistocene and Holocene have had an impact on the distribution of the gazelles in the Southern Levant [51]. All three species are recognized as relatively new post-glacial (Pleistocene) expansions, although fossil evidence of *Gazella* sp. can be found in the Negev as early as the Early Miocene [52]. This study presents the first genetic characterization of the unique Negev Dorcas gazelles. Within this species we found a clear geographic structuring of mitochondrial haplotypes (Fig. 4). Our haplotype network (Cytb+CR) showed a deeper divergence between haplotypes within the population in Israel, compared to the more closely clustered haplotypes from the Saharan populations (Fig. 4). This pattern demonstrates deeper roots for the genetic structure, or conversely habitat fragmentation leading to inter-populations variability. The distance between the haplotypes found in Israel is greater than the distance between the different North African haplotypes, despite the large geographic distances among the Saharan populations. These findings lead us to suggest that southern Levant gazelle population harbors older roots (Fig. 4). Moreover, within our CR dataset we identified a cluster of six specimens from Israel that together with a sample from Sudan and a sample from captivity (King Khalid Wildlife Center) diverged from the main Dorcas clade approx. 1.3mya (Fig. 2). All the specimens from Israel represented in this ancestral cluster originated from the Negev subpopulation, which may be a relict population of a pre-glacial lineage. Unfortunately there were no available sequences for specimens from Egypt or Libya, which may provide more information on the relationships between ancient Southern Levant and North African Dorcas gazelles. We therefore propose a re-examination of the current theory of an African origin for the Dorcas gazelles [37,51], hypothesizing that the Dorcas gazelle may have originated in the Southern Levant and spread to North Africa.

The changes in the environment in the Levant during glacial cycles caused adaptation and speciation of local gazelles, creating a cradle province for *Gazella* [52]. The mountain gazelle, the most northern of the gazelle species in our study, displays genetic patterns consistent with its post-glacial emergence (tMRCA 750kya, Middle Pleistocene, Fig. 2). During the glacial period, this species expanded its range to the south and reached southern Sinai [51] where it most likely shared its habitat with Arabian gazelles. As the weather grew warmer the species withdrew to its present distribution. Since the end of the glacial period the expanding population has not yet accumulated enough mutations along the mitochondrial genome, therefore presenting low diversity levels and a weak phylogeographic structure [i.e. 53,54]. The post-glacial recovery of the species is demonstrated by its high prevalence in archaeological assemblages in the Levant [2,55]. In 2012 Kankilic et al [56] reported on a discovery of a small (n = ~200) mountain gazelle population in Hatay in Turkey, which was found to be closely related to mountain gazelles in Israel based on Cytb phylogenetic analysis.

In the past, the Arabian gazelle’s distribution encompassed the Levant and the Arabian Peninsula, co-existing with Dorcas and mountain gazelles. Recent climatic and anthropogenic changes have limited its distribution to isolated populations, one of which is a relict population in Israel, the acacia gazelle in the Arava. We found that within the Arabian gazelle lineage, based on the mitochondrial markers, all of the studied acacia gazelles formed a cluster together with gazelles from the Farasan Islands and a few captive specimens. This cluster (Farasan Islands and acacia gazelles) differed significantly from the other Arabian gazelles (mainland), with no shared haplotypes between the two groups (Fig. 2). Previous genetic studies, also based on mitochondrial markers, suggested that the population on the Farasan islands might represent an original wild population which was found on the mainland Arabian Peninsula [8,57]. Within this original wild lineage, the acacia gazelles represent a unique group, not sharing haplotypes with any other locality. However we have to note a caveat in this interpretation as most of the published CR sequences of Arabian gazelles originate from captive animals (45 out of 65 Arabian mountain gazelle). It is acknowledged that the origin of captive gazelles is generally uncertain with abundant opportunities for hybridization and mixed ancestry [58], hence inferences based on captive specimens can be misleading. Nevertheless, all of the Farasan samples are reported as originating from the nature reserves on the Islands and not from captive stocks.

### Implications for Species Conservation

The three gazelle species studied here are found in Israel, which has a small geographic area with diverse habitats. Dorcas and mountain gazelles are naturally distributed based on their physiological and ecological adaptations (mountain gazelles in the Mediterranean climate zone and Dorcas gazelles in the semi-arid desert), however at the edge of their distribution, in the Western Negev, both species live sympatrically. Only one report (based on sightings) was found in the literature in which the authors claimed the offspring of such cross-breeding to be unfertile [59]. All the mountain and Dorcas gazelles individuals from the Western Negev clustered with their *a priori* species designation (based on morphological characteristics), no incongruent results between the mtDNA and nDNA markers were found and pairwise comparisons revealed significant differentiation between the two species in this habitat—no hybrids were present in our samples (Figs. 2 & 3).

Acacia gazelles share their protective enclosure with Dorcas gazelles. The genetic analyses applied in this study found a clear separation of the two populations based on both the nuclear and mitochondrial clustering, for all but one individual. Specimen Gd13 was identified as a product of a possible ancient hybridization event: its nuclear DNA profile was found to be ca. 99% of Dorcas descent yet its mitochondrial sequence clustered with acacia haplotypes, though its lineage diverged approximately 500 kya from the other acacia mitochondrial sequences (Fig. 2). Based on these findings we conclude that the hybridization did not occur in recent times but is a relict of a past event, not affecting current population structure. This individual (Gd13) represented the only evidence for mitochondrial introgression in our results and the ancient divergence date for the haplotype implies that the distinctiveness of the acacia gazelle population is not compromised by recent hybridization. Moreover, genetic distance indices revealed high and significant differentiation (Fst) between the Dorcas and acacia gazelle species (Table 1), despite their sharing the same limited habitat.

Each species of gazelle in Israel demonstrated different genetic characteristics. These characteristics suggest different conservation management programs for each species:

### Mountain gazelles

Using both mtDNA and nDNA markers we found low genetic diversity within the mountain gazelle subpopulations with no genetic differences between them, demonstrating an effectively homogeneous population. As noted, we believe the genetic profile is due to the recent population expansion, but also points to ongoing gene flow between the subpopulations. The evidence for gene flow is provided by both the mtDNA markers and the bi-parental microsatellites, which show lack of biogeographic structure and may be a result of natural migration of individuals or occasional translocations as part of species management by INPA [14]. Additionally, the microsatellite AFS describes a population in mutation drift equilibrium [46]. These conclusions eliminate speculation that the small subpopulations of mountain gazelles in Israel are isolated from each other, or under risk of presenting small population genetic effects [5].

The new species status given to the mountain gazelles [8,9], which is supported by our data (Fig. 2, Tables 1 and S4), highlights the local and global uniqueness of the species. Mountain gazelle numbers in Israel today are estimated at about 3000 individuals, forming the largest population known. The recorded ongoing decline in the population size since the 1990s and the increasing pressure from poaching and urbanization requires critical attention, as uncontrolled hunting has already resulted in the extinction of mountain gazelles in Syria, and possibly in Lebanon and Jordan (IUCN). We recommend treating the rare mountain gazelle population in Israel as a single conservation management unit and revising its conservation status to Endangered, owing to the low numbers and restricted distribution. The relevant authorities of Israel and the Palestinian Authority must act immediately to ensure long term viability of the population, which harbors immense significance in global conservation terms, before it is too late.

### Dorcas gazelles

Within the Dorcas population in Israel we found genetic evidence for subpopulation fragmentation, even though the habitat is a wide, relatively open, desert with no known geographic barriers. While their habitat in southern Israel is less populated by humans (in comparison to the mountain gazelle habitat), the type of human activity in this area has greater impact on the Dorcas gazelles. Quarrying, agriculture and military activities in the Negev and Arava cause natural habitat disturbance and fragmentation. To test this hypothesis further would require increasing the sample size with samples collected from the borders of both localities. Management programs should promote ecological corridors and control predation pressure to permit migration of individuals between subpopulations (Negev and Arava).

The genetic diversity measures estimated for the Dorcas gazelles from Israel, including haplotype diversity and nucleotide diversity, were comparable to values reported for other Dorcas gazelle populations from different localities [37,50], indicating that the population in Israel is healthy with a considerable level of genetic diversity. Annual counts of the Dorcas gazelle population in Israel in the last few years revealed relative stability in population size. This is in contrast to the progressive population decline throughout their range in North Africa, with excessive hunting being the major threat to the species, leaving a few fragmented populations in the wild. Therefore the population of Dorcas gazelles in Israel is a valuable source of genetic variability for the species and an important factor in future conservation efforts across their global distribution.

### Acacia gazelles

In this study we compared the genetic profile of this unique population in Israel to different Arabian gazelle populations available in the literature. Our results, based on the mitochondrial control region, revealed acacia gazelles are most closely related to Arabian gazelles from the Farasan Islands, and did not cluster with Arabian gazelles from mainland Saudi Arabia. The same clustering was observed also in a study by Wronski et al (2010). However, in a recent study of nuclear markers acacia gazelle specimens from Israel grouped with mainland gazelles, and Farasan gazelles formed a distinct group by themselves (Lerp 2014). Due to the nature of the markers, we could not compare the nuclear profiles. As mentioned above, most of the mainland gazelle sequences originated from captive specimen with potentially questionable taxonomic status. The distinctive Farasan lineage may represent the original wild haplotype of *Gazella arabica*, not affected by genetic introgression. If this is the case, acacia gazelles share the maternal origins of this unique genetic diversity.

Consequently, conservation measures should treat the acacia gazelle population as its own management unit. Should the need for captive breeding program arises, animals from the mainland or the Farasan islands can be included, however due to the different profiles uncovered in these populations their genetic makeup must be examined thoroughly. The acacia gazelle population in Israel is the only representative of a third gazelle species in Israel and may be the last breeding wild population of acacia gazelles in the world. Additionally, to determine the level, if any, of genetic variation that remains in this single population, we recommend all extant acacia gazelle individuals to be genetically characterized using Next Generation sequencing approach. The information from such a research will help promote the conservation of the extremely rare and genetically unique acacia gazelle population, before it becomes extinct.

For all *Gazella* species, integrating the genetic data into the species’ conservation and management plans must be considered an indispensable step in promoting their survival. To maintain the current levels of genetic diversity, the enforcement of the wildlife protection laws must be enhanced in order to reduce poaching, ecological corridors must be maintained to provide migration between subpopulations, and predation pressure must be controlled to minimize its affect on gazelle recruitment.

## Supporting Information

S1 File The haplotypes of the mitochondrial 12S gene found among the three gazelle species studied in Israel. (DOCX)

S2 File This file contains Supporting Figures. (PDF)

S1 Table Number of samples collected from each gazelle subpopulation and the number of microsatellite and mitochondrial profiles generated for each subpopulation. (DOCX)

S2 Table Information regarding the microsatellite loci used in this study. (DOCX)

S3 Table Pairwise differences among subpopulations of *Gazella* in Israel. (DOCX)

S4 Table Mitochondrial pairwise differences among subpopulations of *Gazella* from Israel, the Arabian Peninsula and Africa. (DOCX)
