# Supplementary material for: Wild Gazelles of the Southern Levant: Genetic Profiling Defines New Conservation Priorities
Source: PLoS One. 2015 Mar 11;10(3):e0116401. doi: 10.1371/journal.pone.0116401 (PMC4356595; doi:10.1371/journal.pone.0116401)
Supplement: S1 File — (DOCX) [file pone.0116401.s001.docx]

**Wild Gazelles of the Southern Levant: genetic profiling defines new conservation priorities**

Lia Hadas, Dalia Hermon, Amizor Boldo, Gal Arieli, Ron Gafny, Roni King and Gila Kahila Bar-Gal

**—Supporting Information File S1—**

**Haplotypes of the mitochondrial 12S gene found among the three gazelle species studied in Israel.**

Mountain gazelle (*Gazella gazella*) - 12S ribosomal RNA gene, Haplotype 1: TTAGCCCTAAACACAAATAATTACATAAACAAAATTATTCGCCAGAGTACTACCGGCAATAGCCTAAAACTCAAAGGACTTGGCGGTGCTTTATACCCTTCTAGAGGAGCCTGTTCTATAATCGATAAACCCCGATAAACCTCACCAATCCTTGCTAATACAGTCTA

Mountain gazelle (*Gazella gazella*) - 12S ribosomal RNA gene, Haplotype 2: TTAGCCCTAAACACAAATAATTACATAAACAAAATTATTCGCCAGAGTACTACCGGCAATAGCCTAAAACTCAAAGGACTTGGCGGTGCTTTATACCCCTCTAGAGGAGCCTGTTCTATAATCGATAAACCCCGATAAACCTCACCAATCCTTGCTAATACAGTCTA

Mountain gazelle (*Gazella gazella*) - 12S ribosomal RNA gene, Haplotype 3: TTAGCCCTAAACACAAATAATTACATAAACAAAATTATTCGCCAGAGTACTACCGGCAATAGCCTAAAACTCAAAGGACTTGGCGGTGCTTTATATCCTTCTAGAGGAGCCTGTTCTATAATCGATAAACCCCGATAAACCTCACCAATCCTTGCTAATACAGTCTA

Dorcas gazelle (*Gazella dorcas*) - 12S ribosomal RNA gene: TTAGCCCTAAACACAAATAATTACATAAACAAAATTATTCGCCAGAGCACTACCGGCAATAGCCTAAAACTCAAAGGACTTGGCGGTGCTTTATACCCTTCTAGAGGAGCCTGTTCTATAATCGATAAACCCCGATAAACCTCACCAATCCTTGCTAATACAGTCTA

Acacia gazelle (*Gazella arabica acaciae*) - 12S ribosomal RNA gene, Haplotype 1: TTAGCCCTAAACACAAATAATTACATAACAAAATTATTCGCCAGAGCACTACCGGCAATAGCCTAAAACTCAAAGGACTTGGCGGTGCTTTATATCCTTCTAGAGGAGCCTGTTCTATAATCGACAAACCCCGATAAACCTCACCAATCCTTGCTAATACAGTCTA

Acacia gazelle (*Gazella arabica acaciae*) - 12S ribosomal RNA gene, Haplotype 2: TTAGCCCTAAACACAAATAATTACATAACAAAATTATTCGCCAGAGCACTACCGGCAATAGCCTAAAACTCAAAGGACTTGGCGGTGCTTTATATCCTTCTAGAGGAGCCTGTTCTATAATCGATAAACCCCGATAAACCTCACCAATCCTTGCTAATACAGTCTA
