# Supplementary material for: Wild Gazelles of the Southern Levant: Genetic Profiling Defines New Conservation Priorities
Source: PLoS One. 2015 Mar 11;10(3):e0116401. doi: 10.1371/journal.pone.0116401 (PMC4356595; doi:10.1371/journal.pone.0116401)
Supplement: S2 File — (PDF) [file pone.0116401.s002.pdf]

Wild Gazelles of the Southern Levant: genetic profiling defines new conservation priorities

Lia Hadas, Dalia Hermon, Amizor Boldo, Gal Arieli, Ron Gafny, Roni King and Gila Kahila Bar-Gal

—Supporting Figures—

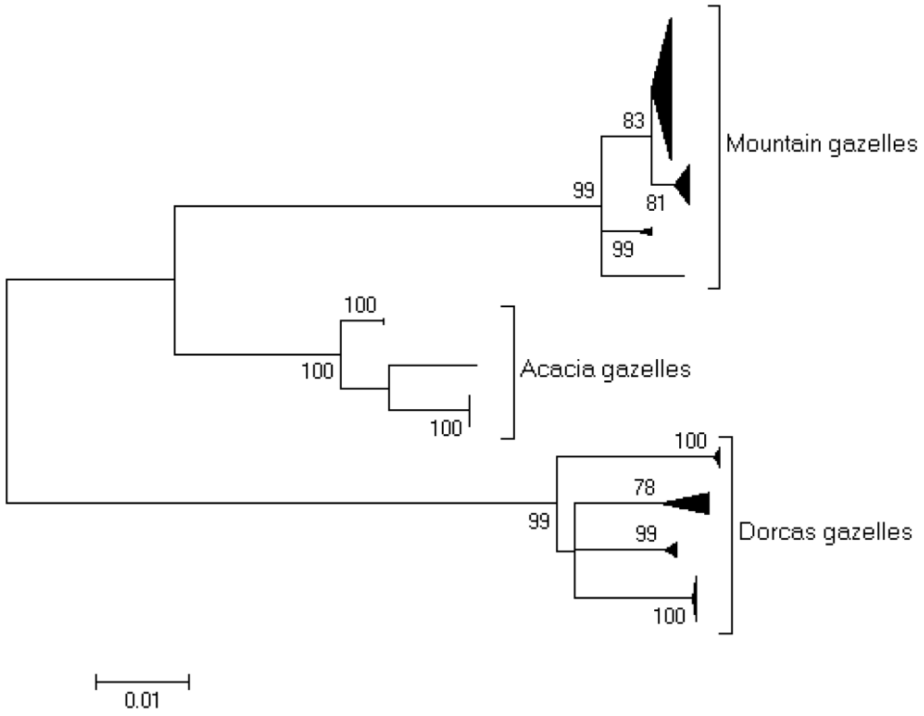

Figure S1. Phylogenetic relationships among gazelle species from Israel. Molecular phylogentic relationships among species of *Gazella* from Israel (n=94) inferred using 1286bp of three concatenated mtDNA sequences (control region, 12S and Cytb). The phylogentic analyses were conducted in MEGA5 [40] using a maximum likelihood method based on the General Time Reversible model + Gamma distributed with Invariant sites (GTR+G+I, 5 discrete gamma categories) [62]. Bootstrap values are shown next to nodes. The phylogenetic analysis supports the separation of the gazelles in Israel into three distinct species: mountain gazelle (*Gazella gazella*), acacia gazelle (*G. arabica acacia*e) and Dorcas gazelle (*Gazella dorcas*). Gd13 is marked with an asterisk.

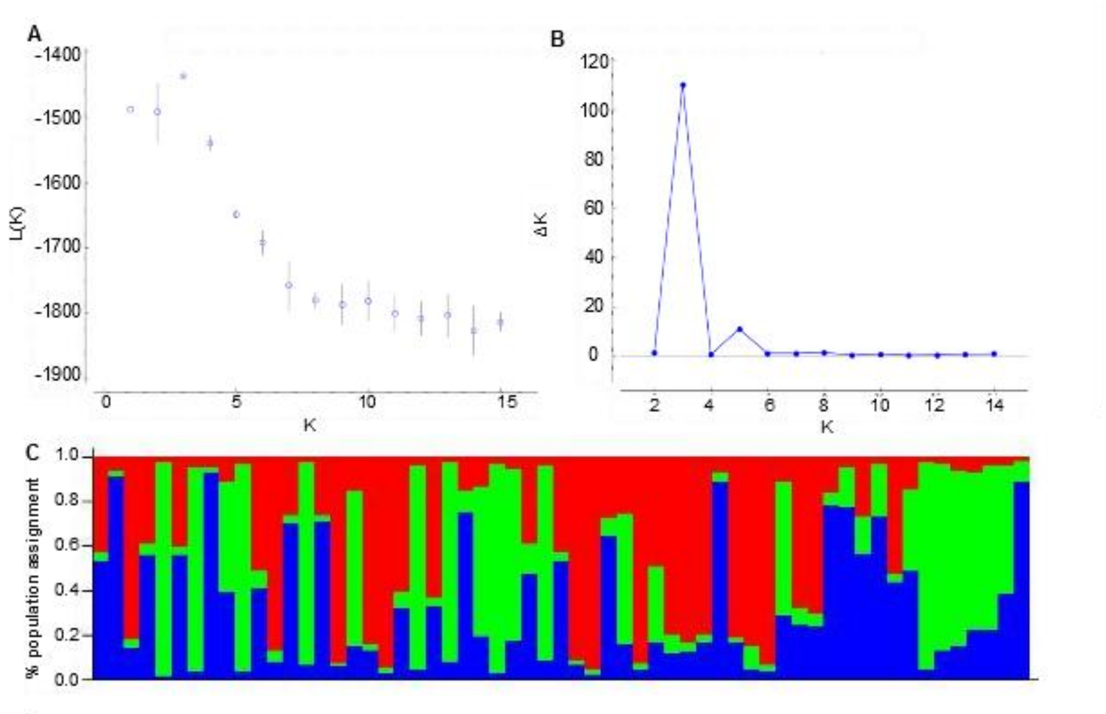

Figure S2. Bayesian clustering analyses for mountain gazelles from Israel. The most probable number of genetic clusters for the mountain gazelle from Israel using the program STRUCTURE (A) Mean L(K)±SD over 5 runs per K as a function of K. (B) ΔK (Evanno et al. 2005) as a function of K. (C) Population assignments to inferred genetic clusters at K = 3 show that the mountain gazelles are divided into three partitions that are not associated with the geographic regions.

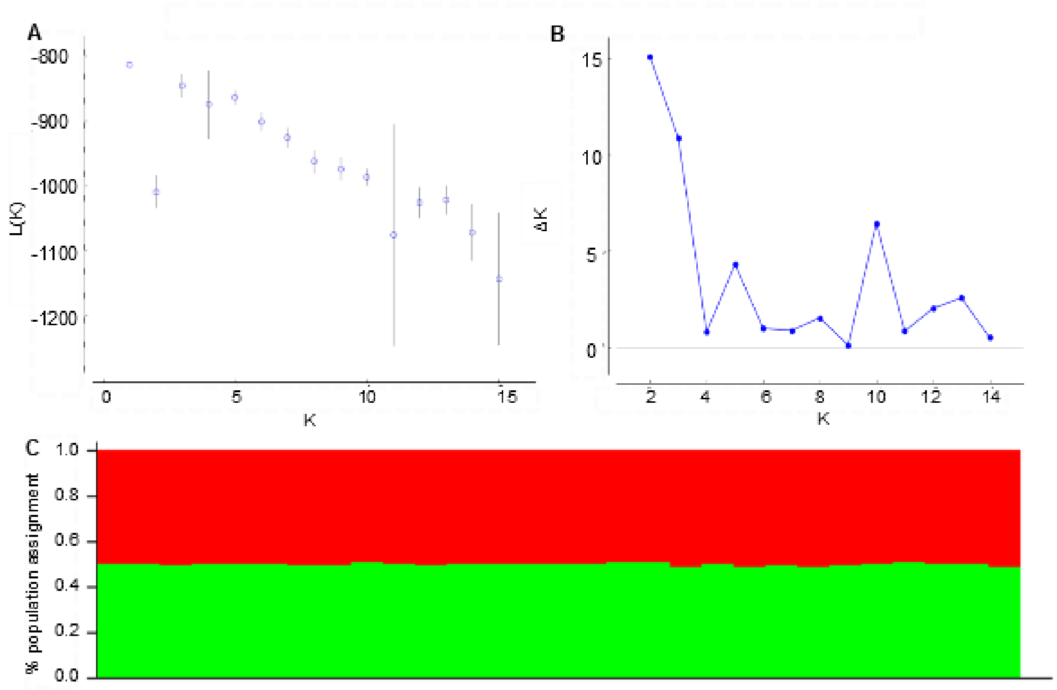

Figure S3. Bayesian clustering analyses for Dorcas gazelle from Israel. The most probable number of genetic clusters for the Dorcas gazelle from Israel using the program STRUCTURE (A) Mean L(K)±SD over 5 runs per K as a function of K. (B) ΔK (Evanno et al. 2005) as a function of K. (C) Population assignments to inferred genetic partitions at K = 2 show that the Dorcas gazelles do not exhibit differentiation between subpopulations.

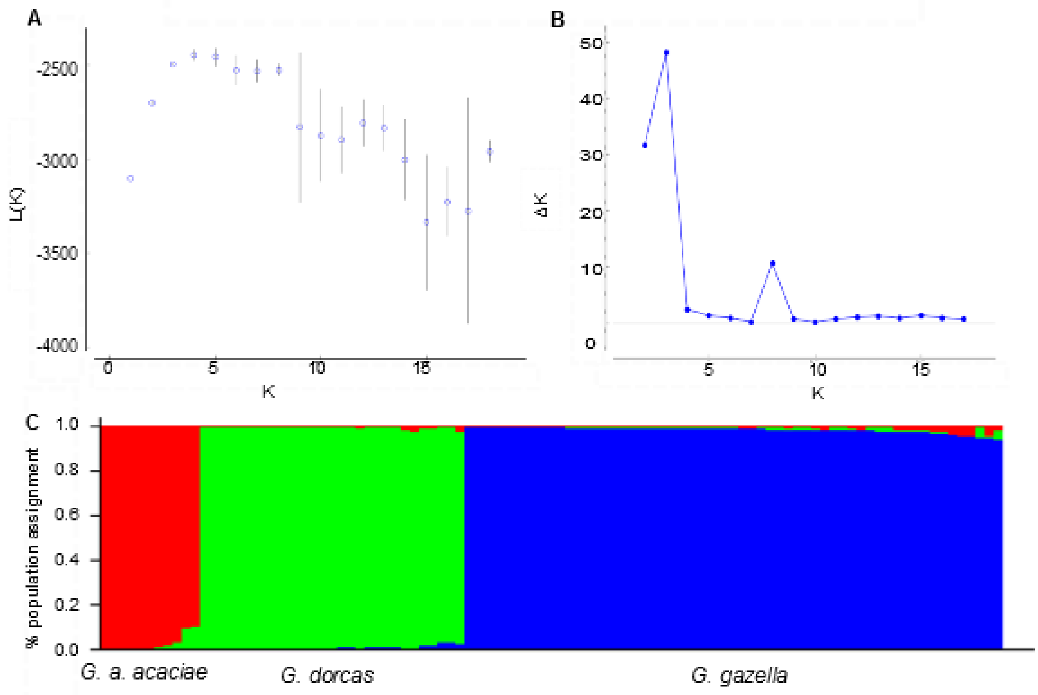

Figure S4. Bayesian clustering analyses for the three gazelle species in Israel (LOCPRIOR). The most probable number of genetic clusters across three gazelle species in Israel using the STRUCTURE program with the LOCPRIOR option. (A) Mean L(K)±SD over 5 runs per K as a function of K. (B) ΔK (Evanno et al. 2005) as a function of K. (C) Population assignments to inferred genetic clusters at K = 3.

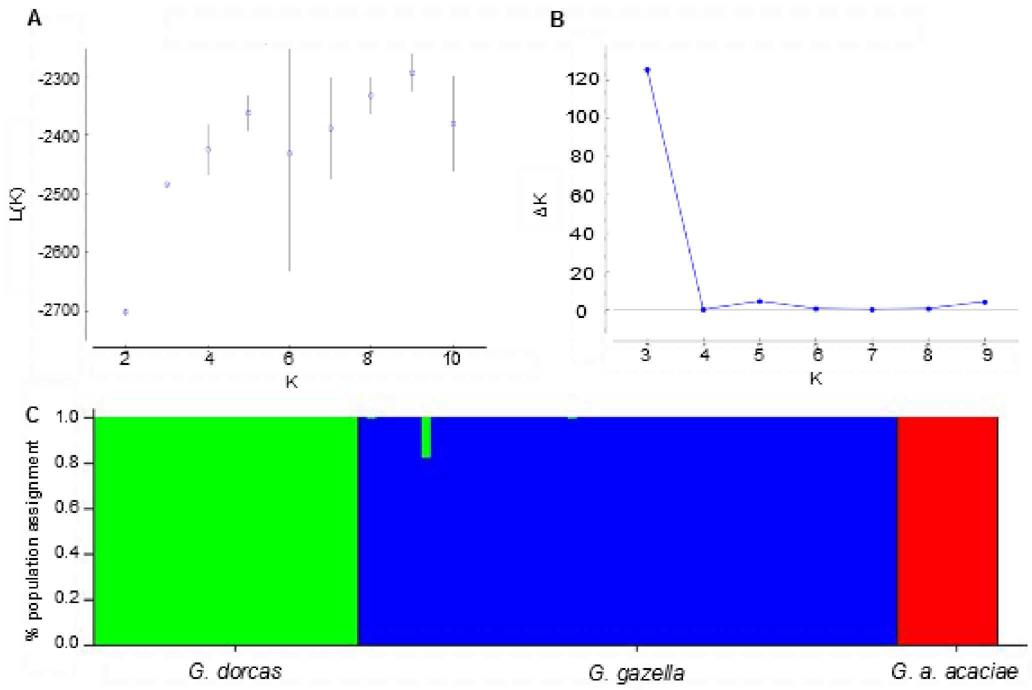

Figure S5. **Bayesian clustering analyses for the three gazelle species in Israel (no admixture).** The most probable number of genetic clusters across three gazelle species in Israel using the STRUCTURE program with the no admixture model. (A) Mean L(K)±SD over 5 runs per K as a function of K. (B) ΔK (Evanno et al. 2005) as a function of K. (C) Population assignments to inferred genetic clusters at K = 3.

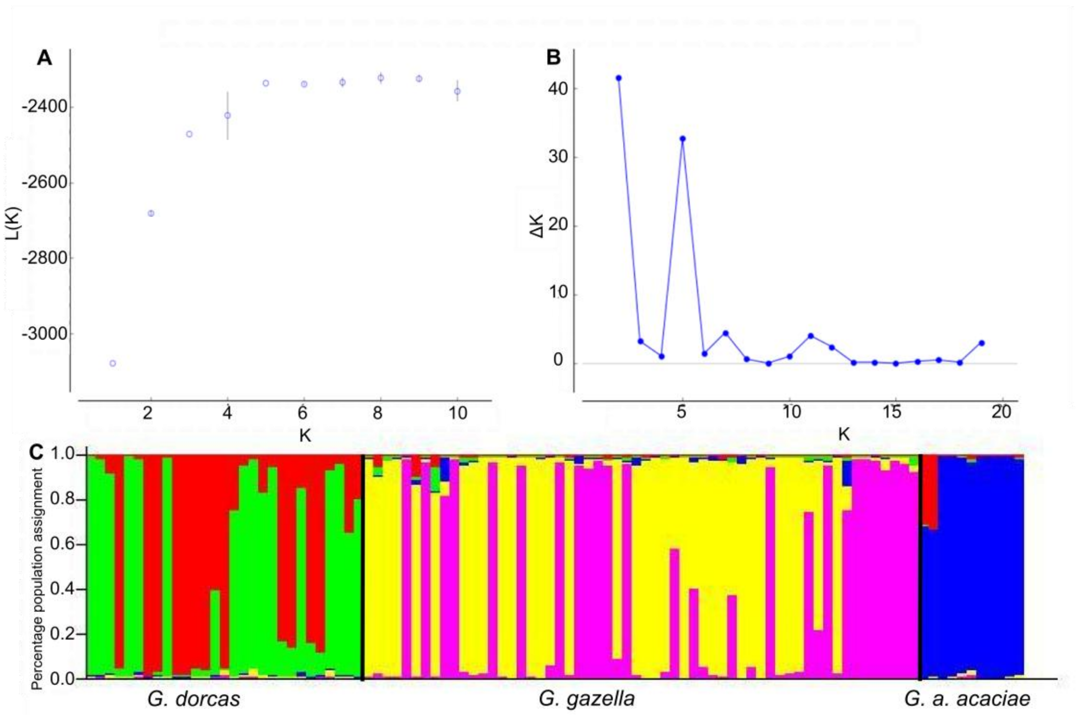

Figure S6. Bayesian clustering analyses for the three gazelle species in Israel

The most probable number of genetic clusters across three gazelle species in Israel using the STRUCTURE program with the standard admixture model. (A) Mean L(K)±SD over 5 runs per K as a function of K. (B) ΔK (Evanno et al. 2005) as a function of K. (C) Percentage population assignments to inferred genetic clusters at K = 5. Two clusters occur among Dorcas gazelles *(Gazella dorcas)* represented by the green and red colors, two clusters occur among mountain gazelles *(Gazella gazella)* represented by the yellow and pink colors, and one cluster occurs among acacia gazelles *(G. a. acacia*) represented by the blue color.

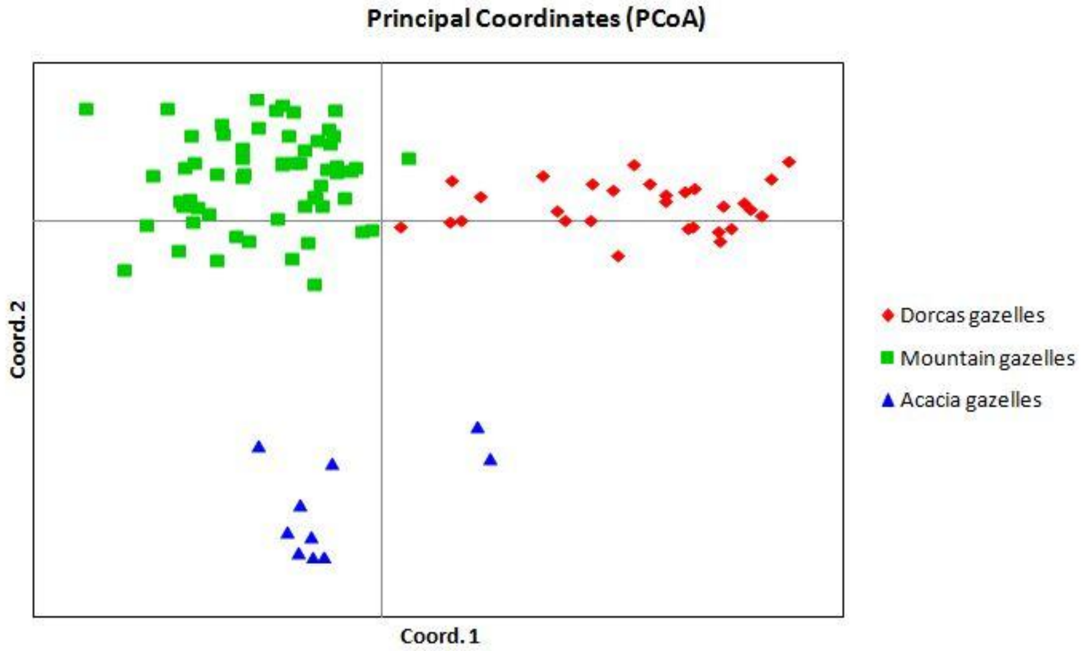

Figure S7. Principal coordinates analysis (PCoA) of the microsatellites data of the three gazelle species in Israel. PCoA Plot was generated with GenAlEx and shows the clustering of each species by itself.
