# Supplementary material for: Wild Gazelles of the Southern Levant: Genetic Profiling Defines New Conservation Priorities
Source: PLoS One. 2015 Mar 11;10(3):e0116401. doi: 10.1371/journal.pone.0116401 (PMC4356595; doi:10.1371/journal.pone.0116401)
Supplement: S1 Table — (DOCX) [file pone.0116401.s003.docx]

**Wild Gazelles of the Southern Levant: genetic profiling defines new conservation priorities**

Lia Hadas, Dalia Hermon, Amizor Boldo, Gal Arieli, Ron Gafny, Roni King and Gila Kahila Bar-Gal

**Table S1** Number of samples collected from each subpopulation and the number of microsatellite and mitochondrial profiles generated for each subpopulation

| **Species** | **Subpopulation** | **Samples** | **STR** | **mtDNA** |
| --- | --- | --- | --- | --- |
| **Mountain gazelles** | Northern | 28 | 27 | 22 |
|  | Central | 24 | 19 | 21 |
|  | Coastal | 6 | 4 | 4 |
|  | Western Negev | 10 | 9 | 7 |
| **Dorcas gazelles** | Arava | 18 | 17 | 15 |
|  | Negev | 11 | 10 | 11 |
|  | Unknown | 3 | 2 | 3 |
| **Acacia gazelles** | Hai-Bar Yotvata Enclosure | 11 | 11 | 11 |
| **Overall** |  | **111** | **99** | **94** |
