# Supplementary material for: Wild Gazelles of the Southern Levant: Genetic Profiling Defines New Conservation Priorities
Source: PLoS One. 2015 Mar 11;10(3):e0116401. doi: 10.1371/journal.pone.0116401 (PMC4356595; doi:10.1371/journal.pone.0116401)
Supplement: S2 Table — (DOCX) [file pone.0116401.s004.docx]

**Wild Gazelles of the Southern Levant: genetic profiling defines new conservation priorities**

Lia Hadas, Dalia Hermon, Amizor Boldo, Gal Arieli, Ron Gafny, Roni King and Gila Kahila Bar-Gal

**Table S2** Information of the microsatellite loci used in the study

| **Locus** | **Reference** | **Multiplex** | **Ta** | **Dorcas gazelle^a^** | | | |  | **Mountain gazelle** | | | |  | **Acacia gazelle^a^** | | | |
| --- | --- | --- | --- | --- | --- | --- | --- | --- | --- | --- | --- | --- | --- | --- | --- | --- | --- |
|  |  |  |  | **Na** | **Ho** | **He** | **PHWE** |  | **Na** | **Ho** | **He** | **PHWE** |  | **Na** | **Ho** | **He** | **PHWE** |
| OarFCB193 | Buchanan and Crawford, 1993 | 1 | 55 | 12 | 0.82 | 0.87 | 0.375 |  | 10 | 0.73 | 0.83 | 0.006 |  | 4 | 0.91 | 0.66 | 0.455 |
| OarFCB48 | Buchanan and Crawford, 1994 | - | 48 | 4 | 0.3 | 0.42 | 0 |  | 6 | 0.19 | 0.2 | 0.089 |  | 3 | 0.18 | 0.31 | 0.007 |
| ETH10 | Solinas-Toldo et al., 1993 | 1 | 55 | 7 | 0.54 | 0.76 | 0.014 |  | 4 | 0.51 | 0.65 | 0.013 |  | 2 | 0.18 | 0.17 | 1 |
| ILSTS029 | Kemp et al., 1995 | - | 52 | 3 | 0.3 | 0.42 | 0 |  | 5 | 0.8 | 0.56 | 0 |  | 2 | 1 | 0.5 | 0.007 |
| BM2113 | Sunden et al., 1993 | 3 | 50 | 5 | 0.57 | 0.68 | 0.213 |  | 11 | 0.71 | 0.8 | 0.035 |  | 3 | 0.1 | 0.55 | 0.001 |
| OarAE54 | Penty et al., 1993 | 2 | 55 | 9 | 0.37 | 0.81 | 0 |  | 12 | 0.68 | 0.8 | 0.029 |  | 2 | 0.09 | 0.09 | 1 |
| TGLA122 | Georges & Massey, 1992 | 1 | 55 | 4 | 0.54 | 0.5 | 0.286 |  | 3 | 0.12 | 0.11 | 1 |  | 1 | N/A | N/A | N/A |
| BM4505 | Bishop et al., 1994 | 2 | 55 | 18 | 0.48 | 0.91 | 0 |  | 11 | 0.46 | 0.69 | 0 |  | 3 | 0.2 | 0.34 | 0.009 |
| INRA40 | Vaiman et al., 1994 | 3 | 50 | 8 | 0.63 | 0.72 | 0.374 |  | 17 | 0.7 | 0.9 | 0 |  | 3 | 0.78 | 0.57 | 0.538 |

Ta=PCR annealing temperature gradient; Na=number of different alleles; Ho=observed heterozygosity; He=expected heterozygosity; pHWE=the probability of Hardy–Weinberg equilibrium.

^a^ Excluding Gd13.
