# Supplementary material for: Wild Gazelles of the Southern Levant: Genetic Profiling Defines New Conservation Priorities
Source: PLoS One. 2015 Mar 11;10(3):e0116401. doi: 10.1371/journal.pone.0116401 (PMC4356595; doi:10.1371/journal.pone.0116401)
Supplement: S3 Table — (DOCX) [file pone.0116401.s005.docx]

**Wild Gazelles of the Southern Levant: genetic profiling defines new conservation priorities**

Lia Hadas, Dalia Hermon, Amizor Boldo, Gal Arieli, Ron Gafny, Roni King and Gila Kahila Bar-Gal

**Table S3** Pairwise differences among subpopulations of *Gazella* in Israel

|  |  | **Dorcas gazelles** | | **Mountain gazelles** | | | | **Acacia gazelles** |
| --- | --- | --- | --- | --- | --- | --- | --- | --- |
|  |  | **Negev** | **Arava** | **Northern** | **Central** | **Coastal** | **W.Negev** |  |
| **Dorcas gazelles** | **Negev** | - | **0.133** | **0.767** | **0.74** | **0.735** | **0.713** | **0.764** |
|  | **Arava** | 0.019 | - | **0.808** | **0.786** | **0.814** | **0.788** | **0.814** |
| **Mountain gazelles** | **Northern** | **0.182** | **0.169** | - | **0.028** | 0.042 | **0.102** | **0.807** |
|  | **Central** | **0.171** | **0.139** | **0.019** | - | 0.038 | 0.006 | **0.775** |
|  | **Coastal** | **0.179** | **0.166** | 0.031 | **0.072** | - | 0.156 | **0.882** |
|  | **W.Negev** | **0.195** | **0.159** | **0.037** | 0.015 | **0.114** | - | **0.82** |
| **Acacia gazelles** | | **0.354** | **0.309** | **0.285** | **0.289** | **0.356** | **0.359** | - |

Pairwise differences Fst based on the 1286bp mitochondrial fragment is given above the diagonal, Fst
