# Supplementary material for: Wild Gazelles of the Southern Levant: Genetic Profiling Defines New Conservation Priorities
Source: PLoS One. 2015 Mar 11;10(3):e0116401. doi: 10.1371/journal.pone.0116401 (PMC4356595; doi:10.1371/journal.pone.0116401)
Supplement: S4 Table — (DOCX) [file pone.0116401.s006.docx]

**Wild Gazelles of the Southern Levant: genetic profiling defines new conservation priorities**

Lia Hadas, Dalia Hermon, Amizor Boldo, Gal Arieli, Ron Gafny, Roni King and Gila Kahila Bar-Gal

**Table S4** Mitochondrial pairwise differences among *Gazella* subpopulations from Israel, the Arabian Peninsula and Africa.

|  |  | **Arabian gazelles** | | | **Dorcas gazelles** | | | | **Mountain gazelles** |
| --- | --- | --- | --- | --- | --- | --- | --- | --- | --- |
|  |  | **Farasan Islands**^a^ | **Arabian Peninsula**^a^ | **Acacia gazelles** | **Israel** | **SC Africa**^b^ | **SE Africa**^b^ | **W Africa**^b^ | **Israel** |
| **Arabian gazelles** | **Farasan Islands**^a^ | - | NA | NA | NA | NA | NA | NA | NA |
|  | **Arabian Peninsula**^a^ | **0.389** | - | **0.489** | **0.832** | **0.870** | **0.804** | **0.873** | **0.948** |
|  | **Acacia gazelles** | **0.371** | **0.516** | - | **0.874** | **0.930** | **0.888** | **0.936** | **0.955** |
| **Dorcas gazelles** | **Israel** | **0.563** | **0.523** | **0.641** | - | **0.485** | **0.209** | **0.339** | **0.923** |
|  | **SC Africa**^b^ | **0.610** | **0.563** | **0.742** | **0.257** | - | 0.118 | **0.455** | **0.966** |
|  | **SE Africa**^b^ | **0.612** | **0.564** | **0.719** | **0.120** | 0.070 | - | **0.225** | **0.947** |
|  | **W Africa**^b^ | **0.617** | **0.581** | **0.765** | **0.320** | **0.320** | **0.346** | - | **0.971** |
| **Mountain Gazelles Israel** | | **0.748** | **0.692** | **0.820** | **0.736** | **0.828** | **0.822** | **0.847** | - |

Fst values based on the CR fragment, 200bp are given below the diagonal, Fst values based on the Cytb fragment, 607bp are given above the diagonal. Bold indicates significance at α=0.05. NA data not available; ^a^ data from [[8](#_ENREF_8)]; ^b^ data from [[35](#_ENREF_35)]
